# ‘Instant’ cusums from a discrete analyser

**DOI:** 10.1155/S1463924685000104

**Published:** 1985

**Authors:** P. Henry, D. C. Ephraim

**Affiliations:** Medical Biochemistry Department Llandough Hospital Cardiff CF61XX UK


Journal of Automatic Chemistry, Volume 7, Number 1 (January–March 1985), pages 46–48

### 'Instant' cusums from a discrete analyser

**P. Henry and D. C. Ephraim**
*Medical Biochemistry Department, Llandough Hospital, Cardiff CF6 1XX, UK*

### Introduction

Quality control is an important feature of today's clinical chemistry laboratory. Most samples are processed in batches, which include quality-control samples. The values obtained for these quality controls can be used in various ways.

The simplest way is to compare the results with the limits of acceptability. Another method is to plot the results graphically: this is more laborious, but it yields more information from the same data, in that it can reveal trends. Judging by the quality-control programs offered by several manufacturers of control sera, this is the way used by many laboratories. A further option is to plot the cumulative sum (cusum) of the results. This is more sensitive still, but is not widely used, probably because of the extra calculations needed, and because few of the standard textbooks on clinical chemistry include any directions on the use of cusums.

Edwards [1] wrote a very helpful practical introduction to the use of cusums, and with microcomputers now being available in many laboratories, extra calculations are no longer a problem. This paper proposes the use of cusums, because more can be deduced about the assays being carried out by using them than can be deduced from a simple graphical plot of the same data.

### Theory

The British Standards Institution's recommendations on the use of cusums [2 and 3] have been followed in both symbols and terminology.

In any method of assessing quality, two measures are needed to define the desired quality: the target value and the variation. When using cusums in clinical chemistry, where individual observations are made from a process under statistical control, the most suitable measure for the target value ($T$) is the mean, and for the variation, the standard error ($\sigma$) calculated from the differences between successive observations, $\delta i$, according to the formula:

$$\sigma = \sqrt{\frac{1}{2(m-1)} \sum_{i=1}^{m-1} \delta_i^2}$$

where $m$ is the number of observations. (In practice, this is usually numerically close to the standard deviation.)

The normal cusum is the sum of the differences of the observations from the mean. However, the mathematical treatment is much simplified if each cusum value is divided by the standard error, hence the cusum:

$$c_i = \sum_{r=1}^{i} \frac{x_i - T}{\sigma}$$

where $x_i$ is the $i$th observation of $x$.

To decide whether the method is still in control, one normally plots a cusum graph, and assesses it using a truncated 'V'-mask, drawn on a moveable transparent sheet of material. The shape of this truncated V determines how many observations are needed before the process is signalled to be out of control, L2 (this should be a small number), and also the average run length when the process is in control, before it is signalled out of control, L1 (which should be very large).

Two parameters are needed to define the shape of the truncated V-mask; one related to the angle between the arms of the V, and the other related to the length of the chord across the V. Following Edwards [1], a V-mask of the shape shown in figure 1 has been used. As only one sample of any quality-control material is used per batch, the V-mask has the length of half the chord (the decision interval), $h = 3{\cdot}4\sigma$, and the slope of the decision line (tan $\phi$), $f = 0{\cdot}82\sigma$ per sample interval. This gives an L2 of five samples and an L1 of about 580, according to the nomogram by Kemp *et al.* [4].

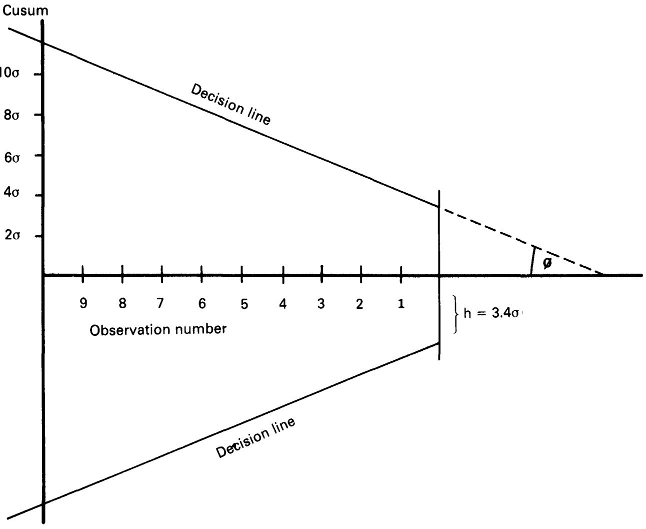

*Figure 1. The truncated V-mask that would be used if the results were plotted manually.*

Instead of judging by eye whether the cusum points lie within the limits or not, it is very easy to calculate with a microcomputer the deviation of all the previous points from the present one ($c_0$), and compare this with the deviation allowed:

deviation generated $= c_0 - c_i$

deviation allowed $= h + if$.

If the decision ratio $c_0 - c_i/h + if$ is greater than unity, or less than minus unity, for any point, then the assay is out of control, high or low.

### Method

Although the concept is applicable to any discrete analyser and microcomputer, the instruments used for the analysis of samples are Beckman ASTRAs, linked to Commodore CBM 4032 microcomputers by Small Systems GP1 1000 interfaces. On the ASTRA 4 (electrolytes, urea and creatinine) one low and one high control are used. On the ASTRA 8 (liver function tests, calcium and enzymes) two pairs of quality-control sera are needed to monitor all the tests. This complicates the program somewhat, but the end result is the same.

Whilst the ASTRA is running, the CBM is dedicated to it in order to generate report forms. When a control sample is signalled, the program switches to the cusum routine. A block diagram of the relevant part of the program is shown in figure 2. Listings of the programs, which occupy about 6K of memory, can be obtained from the authors.

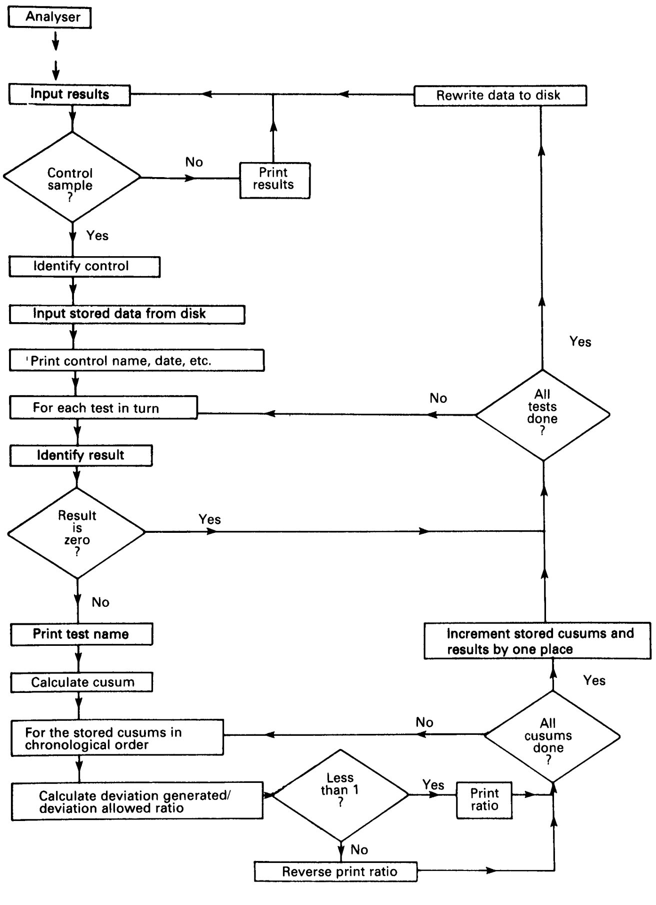

*Figure 2. Flow diagram for printing-out cusum report.*

The 10 previous cusums are stored in files on disk in reverse chronological order for convenience in computing. Only the last 10 observations were used because, in the authors' previous experience, assays out of control were generally signalled as such by one of these points. More points could be used, but this would make the presentation of the result more difficult. An 'edit' routine (not shown) is available to change any of the stored data.

The decision ratio (rounded to one decimal place) is printed-out for each point. If its value is outside the limits, it is emphasized by being printed in reversed characters.

A print-out of a quality-control sample is shown in figure 3. The upper block of figures gives the cusum decision ratios, and the lower block gives the original data from which the corresponding cusums were calculated. The results just obtained are on the right-hand side of the lower block, and the target values are on the left. As is readily evident, the urea assays were out of control.

### Interpretation

As long as there are no figures printed out in reverse, the assays are in control.

If an assay is out of control, the print-out gives some idea of the severity of the problem. If the cusum ratio on the extreme right is printed in reverse, this means that the result for the present batch is grossly different from the target figure, that the run should be terminated and the cause of the error found.

However, if the cusum ratio printed in reverse is not at the extreme right, this means that, over the last few runs, the results have been significantly different from the target figure, according to the cusum statistic. It is still necessary to investigate the cause of the difference but as the errors are not gross and the results for the previous three or four batches have probably been sent out already, it may be reasonable to accept the results. The actual assay figures in the lower block are recorded to help the laboratory director in making such decisions.

The frequency an assay is signalled to be out of control depends on the settings of the mean and the standard error. If these parameters are set from values obtained when the instrument is performing with superb reproducibility, then the assay will often be signalled as being out of control. Conversely, if the parameters are set when the instrument is behaving erratically, then the assay will appear to be in control at times when it is not.

If it is decided that the results for one batch should not be used, then the cusum figures for that batch are deleted, using the 'edit' function.

### Discussion

The system described prints a representation of a cusum graph for several parameters being analysed within seconds of the result being generated in the instrument. Thus it makes available, virtually instantly, information about the status of the analysis, which would otherwise probably only be available retrospectively (if the cusums were plotted manually) or not at all.

The print-out is easy to interpret and includes the original data for the last 10 samples. Thus the system gives the advantages of using cusums (the sensitivity), while

GIB HI

| | | | | | | | | | | | |
|---|---|---|---|---|---|---|---|---|---|---|---|
| SODIUM | | .7 | .6 | .6 | .6 | .5 | .5 | .3 | .3 | .3 | .1 | |
| POTASSIUM | | .1 | .1 | .1 | .2 | .1 | .0 | .0 | .0 | .0 | .0 | |
| BICARB. | | .0 | .0 | .0 | .0 | -.1 | -.2 | -.3 | -.4 | -.3 | -.2 | |
| UREA | | .0 | .0 | .1 | .5 | .4 | .7 | .9 | [illegible] | [illegible] | .7 | |
| CREATININE | | .5 | .4 | .5 | .5 | .4 | .4 | .3 | .3 | .3 | .3 | |
| | TARGET | | | | | | | | | | | ↓ |
| SOD | 154.6 | 157 | 158 | 156 | 156 | 159 | 156 | 158 | 156 | 156 | 157 | 156 |
| POT | 6.92 | 6.9 | 7.0 | 6.9 | 6.9 | 7.0 | 7.0 | 7.0 | 7.0 | 6.9 | 6.9 | 6.9 |
| BIC | 31.8 | 31 | 32 | 32 | 33 | 33 | 33 | 33 | 32 | 30 | 31 | 30 |
| URE | 22.5 | 22.3 | 22.4 | 22.0 | 21.6 | 22.8 | 22.0 | 22.1 | 22.1 | 23.2 | 23.3 | 23.4 |
| CRE | 611 | 619 | 619 | 608 | 616 | 630 | 621 | 617 | 617 | 616 | 616 | 628 |

TRAY 03
ANALYZED AT 1457
8.6.84

*Figure 3. Typical cusum report.*

overcoming the disadvantages which are often encountered (extra calculation, manual plotting of results and loss of original data on the final graph).

When one batch of quality-control material is replaced by another, the change-over is easy. One only has to enter the new values of mean and standard error using the 'edit' function. Because the cusums are standardized by dividing by the standard error, the previous cusums are still valid, and should not be deleted.

The system can be applied to any discrete analyser which has a digital output of results and where the quality-control sera can be identified as such.

The only drawback is that a microcomputer has to be dedicated to the instrument while the analysis is taking place. In our case, we were already using the microcomputer to generate report forms, so this did not apply. As many instruments now have their own microcomputers, we would like to see manufacturers incorporating the idea of 'instant' cusums.

### References

1. Edwards, R. H. W., *Analytical and Clinical Biochemistry*, **17** (1980), 205.
2. *BS 5703: Part 1* (1980).
3. *BS 5703: Part 2* (1980).
4. Kemp, K. W., Nix, A. B. J., Wilson, D. W. and Griffiths, K., *Journal of Endocrinology*, **76** (1978), 203.

### COMPUTING IN CLINICAL LABORATORIES

*Fifth International Conference: Stuttgart, FR Germany (12–14 June 1985)*

The conference programme, which caters for both the general interest of clinical laboratory workers and the special interest of computer scientists, comprises three main sessions at which the following topics will be explored:

*Data-bases*
General background to a data-base
Why one wants a data-base in a laboratory computer system and how it should be justified
How one would set about evaluating laboratory computer systems in terms of their data-base management capabilities.

*Data presentation*
How we should be using colour and graphic displays to achieve maximum impact of data
How to select a graphics terminal
Graphical display of clinical laboratory data.

*Expected developments in laboratory computing*
Expert systems and their application in medicine
A tightening up of the methods of achieving confidentiality in laboratory computer systems
From punch cards to robots.

Each main topic will be developed in expert panel discussions and poster sessions. The proceedings of the conference, including poster presentations, will be published. There will be a trade exhibition including computer systems/peripherals, program demonstrations and trade seminars.

*More information from the Chairman of the Fifth Conference CCL 85, PD Dr Chr. Trendelenburg, Katharinenhospital, Klinisch-Chemisches Institut, Kriegsbergstrasse 60, D 7000 Stuttgart, FR Germany.*

